# Plantar Dislocation of the Middle Cuneiform Bone With Medial Cuneiform Subluxation: A Case Report

**DOI:** 10.7759/cureus.49505

**Published:** 2023-11-27

**Authors:** Erdem Can, Yasar M Dincel

**Affiliations:** 1 Orthopaedics and Traumatology, Ercis State Hospital, Van, TUR; 2 Orthopaedics and Traumatology, Faculty of Medicine, Tekirdağ Namık Kemal University, Tekirdag, TUR

**Keywords:** dislocation, cuneiform, plantar, isolated, neglected

## Abstract

The middle cuneiform is the keystone of the transverse arch and is located between the medial cuneiform and lateral cuneiforms. Isolated middle cuneiform plantar dislocations are rare injuries due to their shape and ligamentous structural connections. A 20-year-old female patient was admitted to the emergency service of another clinic after a 70 kg iron tractor piece fell on her foot 20 days before she was referred to our clinic. Conservative treatment with a short leg circular cast was applied, considering that there was a fracture in the cuneiforms on X-ray imaging. The patient, who was re-evaluated at the second-week follow-up, was referred to our hospital considering the complex injury of the cuneiforms. There was tenderness over the cuneiform in the physical examination. X-ray and computed tomography images of the patient revealed plantar dislocation of the middle cuneiform bone. with subluxation of the medial cuneiform. Open reduction and internal fixation were performed for the middle cuneiform. During the six-month follow-up, the patient had no complaints and was able to do routine work. No lucency or arthritic changes were observed in the X-ray at the 22-month control. Plantar middle cuneiform dislocations are rare and late diagnosis can lead to poor results. This case is presented to emphasize the importance of considering this rare injury and using advanced imaging studies when necessary, considering the mechanism of injury.

## Introduction

The midfoot acts as a bridge between the hindfoot and forefoot, providing stability and flexibility essential for normal walking and other activities. Biomechanically evaluated as three columns, the medial column (navicular, medial cuneiform, first metatarsal) and lateral column (cuboid, fourth and fifth metatarsals) are more mobile, while the middle column (middle cuneiform, lateral cuneiform, second and third metatarsals) is more stable [1]. The stiffness of the middle column acts as a lever in the pushing phase of the gait cycle. Sagittal mobility of the lateral column provides the flexibility needed to walk on uneven ground. In addition, there are limited sliding and rotational movements of the bones during pronation and supination of the foot [2,3].

The middle cuneiform, located between the medial and lateral cuneiforms, is the keystone of the transverse arch. Plantar stabilization is provided with the posterior support of the tibialis posterior ligament in the form of a bone wedge and strong ligament support in the deep plane [2,4]. Dorsal transverse ligaments contribute to the stabilization of the middle cuneiform as well as the support of the interosseous ligaments. Bone receives its nutrition from the medial and lateral surfaces as well as from the dorsal surface [2,4]. Due to its shape and ligamentous structural connections, isolated middle cuneiform plantar dislocations are rare injuries. According to our knowledge, seven cases have been reported in the literature [1-3,5]. In addition to these cases in the literature, cases showing delayed admission with good results, as in our case, are limited. In our case, in addition to the dislocation of the plantar middle cuneiform, subluxation of the medial cuneiform is also being observed, and to the best of our knowledge, no such case has been reported in the literature.

We present a report assessing the significance, diagnosis, consequences, and surgical management of these injuries.

## Case presentation

**Figure 1:**
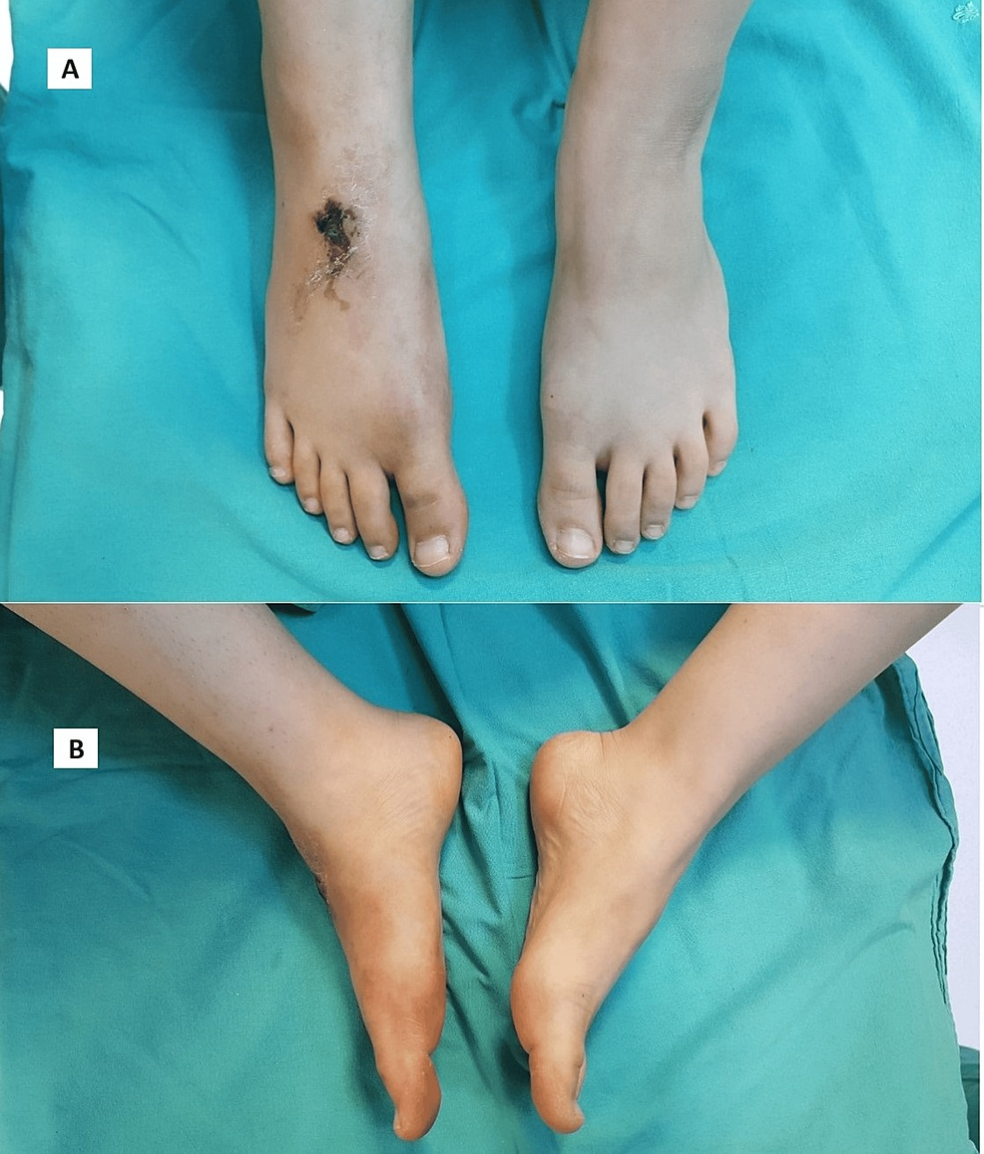
Images of the injured foot at first admission A: The patient presents with ecchymosis and a crusted wound on the right foot. B: Examination shows a medial arch collapse in the right foot.

A 20-year-old female patient was admitted to the emergency service of another clinic after a 70 kg iron tractor piece fell on her foot 20 days before she was referred to our clinic. Conservative treatment was applied with a short leg circular cast, considering the fracture in the cuneiforms visible on X-ray imaging. The patient, who was re-evaluated at the second-week follow-up, was referred to our hospital considering the complex injury of the cuneiforms. There was no chronic disease in the patient's medical history, and there was no history of smoking or alcohol use. During the medical evaluation conducted upon her admission to our hospital, there was a crusted wound on the back of the foot and there was an ecchymosis with discoloration extending to the medial side of the foot. The vascular and sensory assessment of the foot exhibited natural results, including the presence of a dorsalis pedis pulse and preservation of normal deep peroneal function (Figure 1).

**Figure 2:**
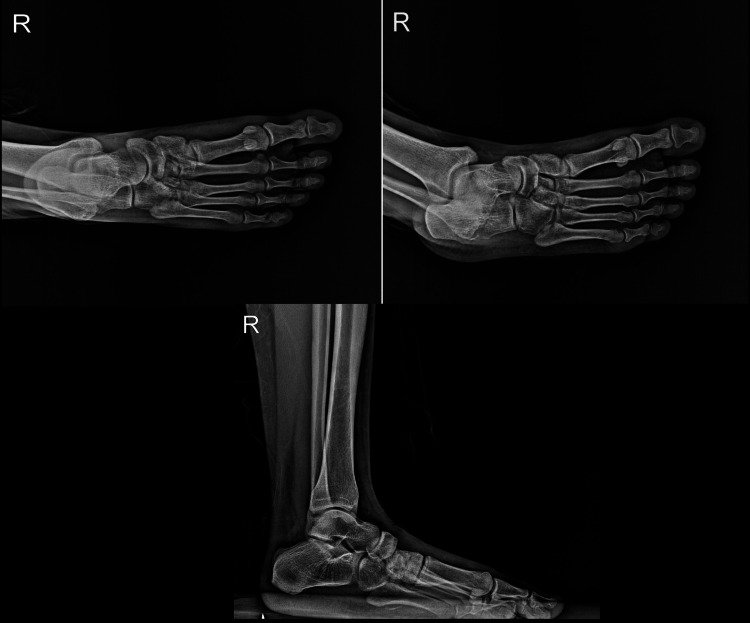
Pre-operative X-ray images Cuneiform irregularity is visible on AP and oblique views, while the lateral radiograph shows partial longitudinal arch collapse.

**Figure 3:**
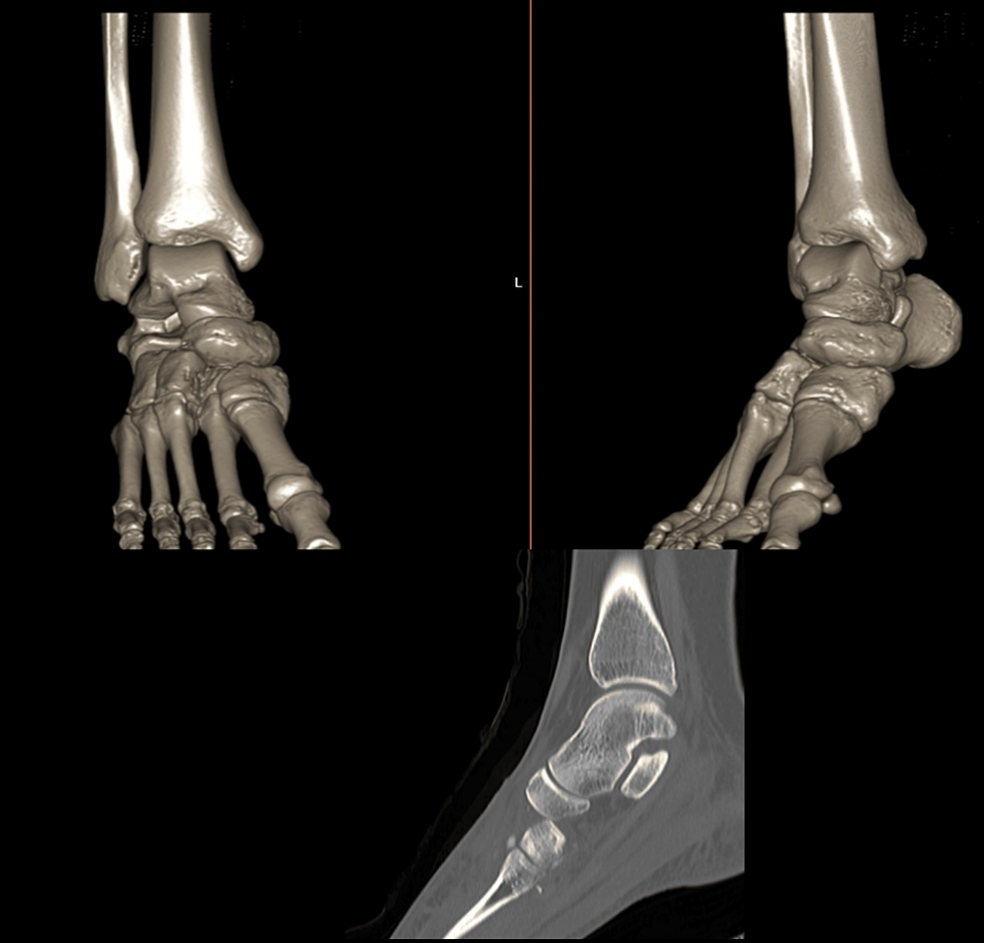
Pre-operative CT scan images of the patient

The patient presented with mild swelling in the midfoot area, along with a slight depression of the medial arch in comparison to the contralateral side. Tenderness was noted over the cuneiform, and the patient experienced pain during foot eversion and inversion. X-ray and computed tomography (CT) images of the patient were performed. The X-ray images revealed irregularities in the cuneiforms, but the middle cuneiform dislocation could not be distinguished. Additionally, the imaging revealed that there was arch continuity with partial collapse of the longitudinal arch when compared to the opposite foot (Figure 2). Middle cuneiform plantar dislocation and slight medial cuneiform subluxation were detected on CT imaging, and it was notably visible in the 3D imaging as well (Figure 3).

**Figure 4:**
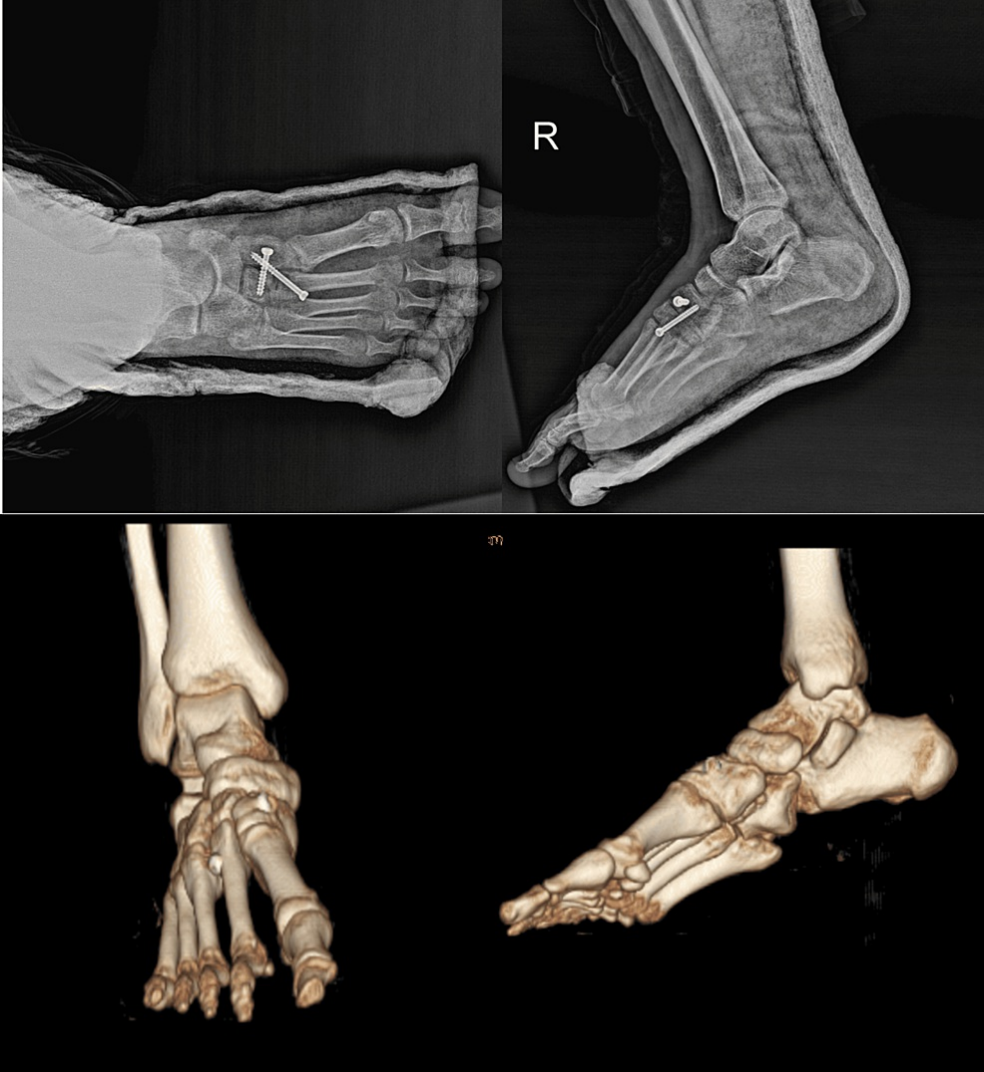
Post-operative radiographs and 3D images, demonstrating successful reduction

The patient was not a candidate for closed reduction, so it was decided to proceed with an open reduction. A longitudinal incision was made from the dorsal aspect of the existing wound and extended to expose the base of the second metatarsal. Fibrous soft tissue appeared to obscure the area where the middle cuneiform should be located at the proximal of the second metatarsal. The medial cuneiform was subluxated and reduced spontaneously upon using a blunt Hohmann retractor as a lever to reduce the middle cuneiform. It was noted that the cuneiform bones, which exhibited irregularities before reduction, showed proper alignment and compatibility after reduction. Considering the patient's age and activity level, and with evaluation of the reduction quality, it was decided to fix the bones and secure the reduction with cannulated screws. Middle cuneiform was fixed to medial cuneiform and 2nd metatarsal with 4.5 mm cancellous cannulated screws (TST, Istanbul, Turkey) (Figure 4).

**Figure 5:**
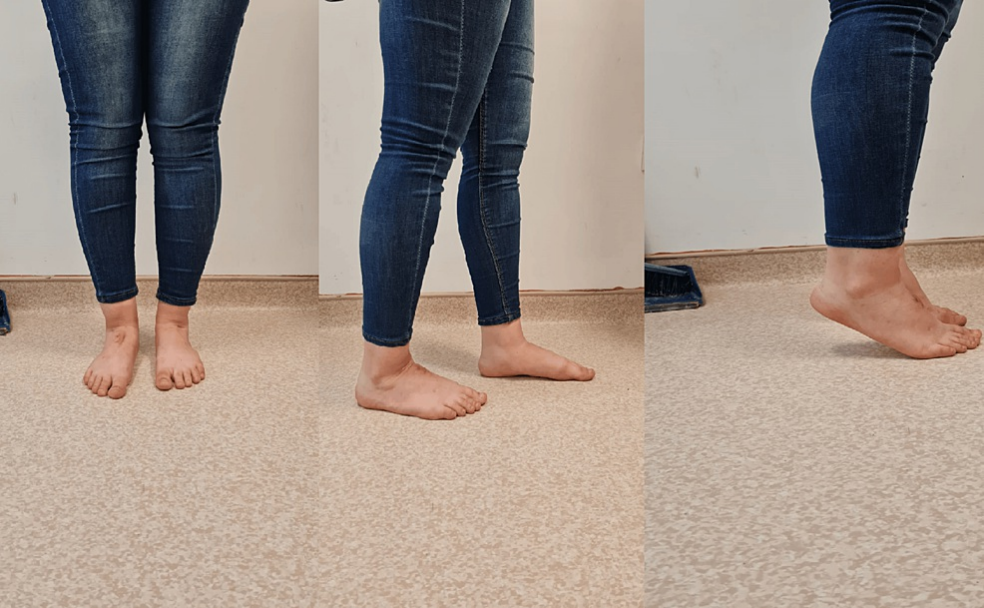
Clinical images of the patient at the sixth-month follow-up

**Figure 6:**
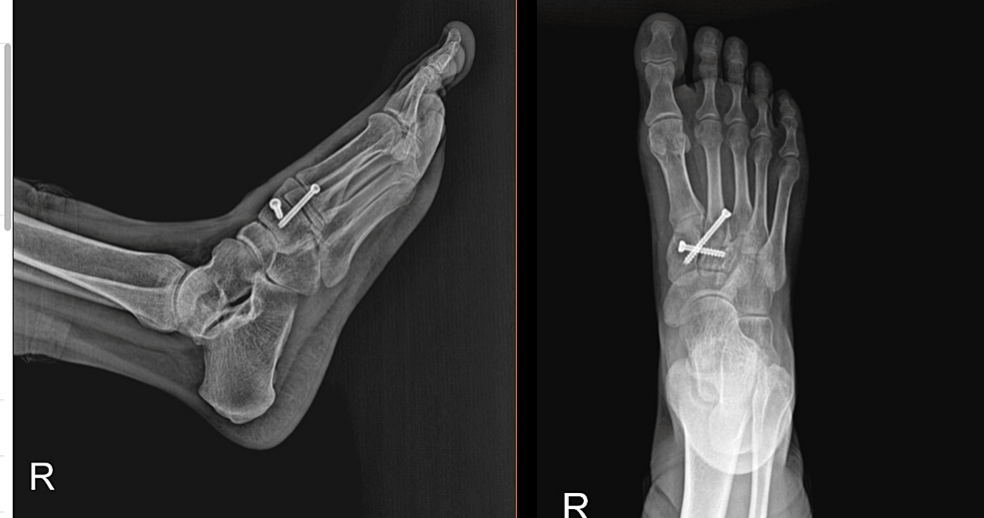
AP and lateral X-rays at the 22nd-month follow-up The AP and lateral radiographs do not indicate any signs of lucency or tarsometatarsal and intermetatarsal arthrosis. AP: Anteroposterior

In the fluoroscopy control, the alignment was found to be anatomical and the stability was good after fixation. During the sixth-month follow-up, the patient had no complaints and was able to do routine work, including heavy work as a farmer (Figure 5). No obvious lucency was observed in the X-ray during the 22-month outpatient clinical check-up, and no arthritic changes were found based on the mid-term results (Figure 6).

The patient was questioned and examined with The American Orthopaedic Foot and Ankle Society (AOFAS) Ankle-Hindfoot questionnaire, and her AOFAS score at 22 months was 88%. The patient was informed that her condition could be published, and her written consent was obtained.

## Discussion

The foot must be both flexible to provide balance on uneven surfaces and stable enough to bear weight. During the walking cycle, the shape of the foot also changes [1].

The middle cuneiform and the second metatarsal are the keystones of the foot's arch. Due to these important positions, the arch of the foot may be affected as a result of injuries to these bones, and arthrosis and degeneration may develop in the midfoot joints with the development of arch collapse [2].

The middle cuneiform articulates with medial and lateral cuneiforms located at the top of the arch of the foot and also with navicular bone and the second metatarsal. The arch is supported by interosseous, dorsal, and intermediate ligaments as well as the posterior tibial tendon and plantar fascia [2,4]. Since the middle cuneiform base is dorsally oriented and wedge-shaped, with the ligament support in the plantar region; the bone is prone to dorsal dislocation [2,3]. Sometimes dislocations can occur indirectly, as a Lisfranc variant, but often this injury is caused by direct trauma [3].

Plantar middle cuneiform dislocations are more difficult to reduce. In a study in which three cases were reported, the results were reported to be poor. Cases with good results after plantar dislocation are limited [3,5]. In addition, a case with a nine-month late presentation was presented in the literature, in which the cuneiform was excised and the surgical results were not comparable [6]. In our case, an injury was initially overlooked, and the reason for referral was the irregularity of the bone alignment, with no clear diagnosis. Despite the delayed referral in our case, the results were acceptable, and the patient was able to carry on with her daily life, performing heavy work as a farmer. Delayed reduction of dislocations can cause arthrosis, and our case presented a result that did not go into arthrosis with its current results. Although it is obvious that longer-term results are needed for arthritis, this may be instructive for mid-term results. Our case report provides the literature with data that satisfactory results can be obtained with primary reduction and fixation, even in late presentation, which strengthens the argument that arthrodesis should remain the last option.

## Conclusions

Dislocations of the plantar medial cuneiform with plantar medial cuneiform subluxation are a rare condition. As observed in our case, these dislocations may go unnoticed, leading to a delay in treatment and, consequently, poor results. To ensure prompt diagnosis, it is essential to consider the mechanism of trauma, conduct a thorough physical examination, and utilize CT imaging in case of any uncertainty.
